# Processes for engaging and retaining women who are experiencing adversity in longitudinal health services research

**DOI:** 10.1186/s12913-019-4698-5

**Published:** 2019-11-14

**Authors:** Anna Price, Hannah Bryson, Ashlee Smith, Fiona Mensah, Sharon Goldfeld

**Affiliations:** 1 Centre for Community Child Health, The Royal Children’s Hospital, Parkville, Vic 3052 Australia; 2 Population Health, Murdoch Children’s Research Institute, Parkville, Vic 3052 Australia; 3 Department of Paediatrics, The University of Melbourne, Parkville, Vic 3052 Australia; 4 Clinical Sciences and Biostatistics Unit, Murdoch Children’s Research Institute, The Royal Children’s Hospital, Parkville, Vic 3052 Australia

**Keywords:** Quality improvement, Health services research, Vulnerable, Recruitment, Follow-up studies

## Abstract

**Background:**

Women and families experiencing socioeconomic and psychosocial adversity are the least likely to access health care but most likely to benefit. For health services to effectively meet the needs of individuals experiencing adversity, research involving the health services must be truly representative. However, individuals experiencing adversity are typically excluded from or underrepresented in health services research. This paper reports on the implementation of a quality improvement approach designed to support recruitment and retention of pregnant women experiencing adversity in a longitudinal, health services randomized controlled trial (“right@home”).

**Methods:**

right@home recruited Australian women from 10 public maternity hospitals across the states of Victoria and Tasmania who were experiencing adversity (≥2 risk factors on screening survey). Regular follow-up assessments were conducted by phone or face-to-face to child age 2 years. Research processes were designed taking heed of previous research demonstrating effective strategies for recruiting and retaining minority groups (e.g. piloting the recruitment process; recruiting via the health service providing care to the subgroup; remunerating participants); however, we were concerned that important information was missing. Therefore, once recruitment began, we conducted a continuous evaluation of the research processes, testing and implementing changes to processes or new strategies to maximize recruitment and retention (e.g. using a suite of strategies to maintain contact with families, using flexible data collection methods, obtaining consent for data linkage for future health and education data).

**Results:**

right@home enrolled a large cohort of women (*N* = 722) experiencing high levels of adversity according to socioeconomic status and psychosocial risk factors, and achieved excellent retention (83% completion at 2 years). Most strategies appeared to increase recruitment and retention. All required additional time from the research team to develop and test, and some required extra funding, which ranged from minor (e.g. printing) to substantial (e.g. salaries, remuneration).

**Conclusions:**

By taking a quality improvement approach, supported by sufficient resourcing and flexible research processes, it is possible to recruit and retain a large cohort of women experiencing adversity who are typically missed or lost from longitudinal research.

## Background

Recruitment and retention of participants in large-scale, longitudinal, health services research can be complex and challenging, especially when participants are selected for their experience of socioeconomic or psychosocial adversities [1]. Individuals experiencing adversity have the greatest need for health services and supports but are the least able to access them (described as the “inverse care law”) [2]. Reasons for this include cost and an individual’s ability to identify their own needs and seek and obtain services, and these barriers can be compounded by a mistrust of or difficulty relating to the system [3]. Given that individuals experiencing adversity are less likely to interact with health services, researchers can struggle to make contact with eligible individuals in the first place, limiting opportunities for participation.

When it is possible to make contact and invite participation, the artificiality of research design can discourage involvement. Research activities such as providing informed consent, travel and related costs to attend assessments, and finding time to complete assessments, can all be burdensome. Committing to research over years can be difficult for individuals with less stability in their lives or less confidence in, or literacy or familiarity with, health services [1]. However, to determine whether services can be accessed by and improve outcomes for these individuals, health services research must be truly representative of the population being studied. Otherwise, there is the potential for research funding to be wasted on evaluating services that are either inaccessible or ineffective for the individuals most in need of support [1].

In 2012, recognizing the substantial adversity and poor outcomes experienced by some Australian families, the Victorian and Tasmanian governments together with philanthropy funded the “right@home” partnership to develop and evaluate the largest multi-site, multi-state, Australian randomized controlled trial of nurse home visiting, offered from pregnancy to child age 2 years [4]. Designed for delivery via the child and family (CFH) service that is freely available to all Australian families, right@home aimed to improve the learning and development of children born to women selected for their experience of adversity. We designed the research processes for right@home using the strategies identified by previous research for maximizing recruitment and retention with minority groups (such as ethnic/cultural minorities and individuals with reduced socioeconomic resources) [1]. These strategies included identifying the venues or methods that reach the population of interest [5]; using researcher-led instead of clinician-led recruitment to reduce gatekeeping or selection bias [6]; piloting recruitment processes for acceptability and feasibility [1]; collecting informed consent and data in-person to help support individuals with low literacy [7]; and acknowledging the time and cost burdens of research participation by offering remuneration [1].

Although these processes appeared necessary, we were concerned that they may not be sufficient, and therefore aimed to investigate whether a quality improvement approach to implementing and testing the research processes could maximize recruitment and retention of women in the trial. The intention was not to evaluate whether specific processes worked on their own. Rather we aimed to evaluate whether taking a quality improvement approach using multiple, flexible processes could achieve (i) the target sample size (*N* = 714, see Protocol for calculation [4]) and (ii) prevent loss-to-follow-up as far as possible by attempting to treat every enrolled participant as essential to the trial’s validity.

## Methods

The right@home RCT (ISRCTN89962120, 10.1186/ISRCTN89962120) is unique in the Australian research landscape because it prioritized the 5–7% of pregnant women experiencing greatest adversity and was delivered via the existing CFH service. Methods are previously described in the published Protocol [4]. Briefly, researchers recruited pregnant women from the waiting rooms of antenatal clinics in 10 public maternity hospitals across Victoria and Tasmania, inviting them to complete a 10-item brief risk factor (BRF) survey [8]. Women with 2 or more risk factors (plus additional eligibility criteria, see Additional file 1: Table S1) were invited into the trial. Interested and eligible women were visited in their homes by researchers who collected informed consent and conducted a comprehensive baseline questionnaire before random allocation.

The intervention comprised 25 home visits from pregnancy to 2 years, focusing on parent care of the child, responsivity to the child, and providing a good quality home learning environment. The standard CFH service provided the comparator (control), comprising around 6–9 predominately clinic-based appointments. Researchers followed-up with women in both groups via phone interviews (approximately 30 min) when children were 6 weeks, 6 months and 18 months old, and via home-based interviews (approximately 2 h) when children were 1 and 2 years of age. Women’s postcodes were linked with the national Socio-Economic Indexes for Areas Index of Relative Disadvantage (SEIFA); lower scores indicate greater adversity [9].

The research processes for recruitment and retention – the focus of this paper – were designed based on the aforementioned literature plus input from the program developers. For example, we piloted the recruitment processes, [8] which helped us understand how the antenatal clinics ran; build relationships with clinic staff; assess whether the BRF survey was a useful measure for identifying eligibility; gauge women’s interest in participating in the larger RCT; and decide how to remunerate women. Once recruitment began, we implemented a quality improvement approach, defined as a weekly [5] evaluation of how the research processes were supporting or hindering recruitment and retention. For this, informal feedback – termed anecdotal in the Table 1 – was sought from field researchers, participants and other collaborators (e.g. hospital clinic staff), on perceived barriers to recruitment and retention, and strategies to overcome them. Feedback was summarized and discussed at weekly investigator meetings. Based on consensus, the team retained processes that appeared to contribute to increasing the sample size and retention, and discarded those that did not. As the research team were blinded to randomization status until all 2 year data were collected, our processes were applied to both trial arms without differentiation.

**Table 1:** Barriers anticipated or encountered, strategies implemented, and implications of these strategies

Barrier	Strategy	Description	Outcome	Implications
Sampling frame				
Anticipated difficulty accessing the population of interest [1] (pregnant women experiencing adversity)	Venue-based sampling [10]	We identified a health service (antenatal clinics at public hospitals) that a large proportion of the population would visit, and that researchers could recruit at. We worked with state government partners to identify eligible hospitals serving women where both the postcode-level disadvantage, [9] and children’s developmental vulnerability according to census-level data, [11] were high. We met with antenatal clinic managers to understand client demographics (i.e. level of adversity, birth rates, how close patients live to hospital), and how clinics were run (i.e. triage, wait times, new/high risk/review appointments), to decide which clinics to recruit from.	We successfully recruited a large cohort of women experiencing adversity. Of the 5586 women who completed the BRF survey (see Figure), the average SEIFA was 972.6 (Australian average is 1000; lower scores reflect greater adversity). Of these, 78.3% lived in postcodes from SEIFA quintiles 1–3 (experiencing greatest adversity), compared with 60% nationally [12]. SEIFA was 953.6 for 1427 women who were eligible for the RCT, compared with 979.1 for 4159 ineligible women.^a^	Identifying trial sites that provided care for a high prevalence of women experiencing socioeconomic adversity was a key factor in recruiting the large cohort. Initiating relationships with the antenatal clinic managers was aided by the state government partners. The scoping work to identify suitable clinics, build relationships and establish processes with clinic staff took approximately 6 months from first contact to commencing recruitment, which included obtaining HREC approval. Face-to-face meetings with clinic management were crucial for developing a partnership and processes for data collection.
Anticipated selection bias if recruitment conducted by clinic staff [6]	Researcher-led recruitment	We used researcher- rather than clinician-led recruitment to minimize burden on the already-busy antenatal clinic staff and avoid the possibility of gatekeeping or cherry-picking by clinician-recruiters.	Recruitment by researchers in antenatal clinic waiting rooms was acceptable to women and feasible: of 6977 eligible women surveyed, 5586 (80.1%) completed the survey; 468 (6.7%) started but left the waiting room before finishing (e.g. for an appointment); and 923 (13.2%) declined. These numbers are consistent with the findings of the recruitment pilot study (described below) [8].	There were no data available for the 20% of non-responding women to compare their demographics and levels of adversity with participating women, so this may have implications for the generalizability of the RCT findings.
Recruitment and obtaining consent				
Potential for infeasible or unacceptable recruitment processes [1]	Pilot recruitment processes	Recruitment processes were piloted with all women (*N* = 189) attending antenatal clinics at 2 participating hospitals on 3 consecutive days.	166/186 (89%) of eligible women completed the survey. The high response and zero missing data demonstrated feasibility and acceptability of the recruitment process [8].	The pilot was invaluable for planning recruitment for the RCT. Strong research-clinic relationships were forged using this initial collaborative process, and the pilot clinics acted as champion sites for the larger RCT.
Recruitment started slower than necessary to achieve sample size	Expand catchment areas	We worked with clinic managers to identify additional clinics that researchers could recruit from.	61/722 (8.4%) additional participants were recruited from 3 additional clinics, which contributed to recruiting the required sample size [4].	HREC approval was required for protocol modification. Additional meetings and training between research team and clinic staff were necessary.
	Extend recruitment phase	The number of eligible women attending clinics was lower than anticipated based on previous annual birth rates. The recruitment phase was extended by 6 months (coordinated by trial directors, the Australian Research Alliance for Children and Youth – see Acknowledgements)	198/722 (27.4%) additional participants were recruited, which contributed to recruiting the required sample size [4].	Substantial additional funding was required to extend the research and intervention salaries; changes to contracts with staff and local governance organizations; changes to contracts with funding and government partners. The slower than anticipated recruitment rate had the greatest potential for negatively impacting the RCT’s statistical power and generalizability. Extending the trial dates and funding therefore made the greatest impact on the trial’s eventual validity.
	Recruitment flyer available at clinics	Flyers were left in participating hospital clinics, and some additional clinics of General Practitioners (primary care doctors providing shared antenatal care) for the duration of recruitment period. Interested women could contact the project coordinator and complete the BRF survey by phone.	19 women contacted the research team after seeing a flyer and completed the BRF survey by phone. 5/19 (26%) enrolled in the RCT (1 declined, 13 were ineligible), contributing 0.7% of the final enrolled cohort of *N* = 722.	Time was required to design flyer, obtain HREC approval for protocol modifications, and conduct informed consent and complete the BRF survey over the phone with women, plus printing costs.
Anticipated low literacy [7]	Face-to-face recruitment	Researchers offered to go through the recruitment materials (information statement and consent, and survey) verbally with each woman.	Women requested a verbal explanation infrequently at recruitment (no data collected/available to describe numbers). Note: women with insufficient English to participate in face-to-face interviews were excluded from the trial (667/9511 (7%) when first approached in clinic waiting rooms) [12].	The research findings may not generalize to women with insufficient English to participate. In addition, women with low literacy may be overrepresented in the 923/6977 (13%) women who declined to complete the recruitment survey [12].
	Readability	All printed materials were written at a Grade 6 level or less (primary/elementary school).		
Recontacting eligible women for formal enrolment	On-the-spot bookings	Recruitment began with a staggered approach across the participating sites. For the first 2 months, when recruitment was taking place at 4 Victorian sites, researchers invited women to complete the BRF, recorded the details of eligible and interested women, and attempted to recontact them in the following days to book the enrolment home visit. However, recontacting women proved difficult. The scheduling process was changed to book the enrolment visit with women on-the-spot, once the BRF survey was complete.	For the first 2 months, across the 4 initial Victorian sites, 61/172 (35%) eligible women enrolled, and the average time between completing the BRF survey and the enrolment home visit was 22 days (range 2–112). For an equivalent 2-month period once on-the-spot bookings were introduced at the same 4 sites, 48/102 (47%) eligible women enrolled with an average time of 18 days (range 0–91) between BRF survey completion and the scheduled enrolment visit.	On-the-spot bookings required an online, confidential, real-time calendar that was accessible to all researchers via internet-connected tablets (which was the primary method for data collection for right@home). For this study, the calendar was custom-designed by the research institute’s IT department. Funding was necessary for the IT contract, tablets and plans.
Inaccessible or intimidating study information	Appealing, promotional study materials [5]	Designing study materials to be appealing and promotional. These included: • Giving women an enrolment pack that included a magnetized card with the study details, contact information and enrolment appointment details; • Adding a simple, 1-page colorful flyer to the enrolment pack to precede the lengthy information statement and consent form; • Increasing the font and spacing on the detailed information statement and consent form; • Hanging simple posters and leaving flyers in the waiting rooms that described recruitment; • Placing a placard with the researcher’s name “[Name] is recruiting today” at reception desks, and asking reception staff to tell women about recruitment and encourage interested women to speak to the researcher.	There were no data to describe the usefulness or otherwise of these strategies; however, anecdotally, participants told the researchers that they used and referred to the magnetized cards frequently and, when approached by the researchers in clinic, women often said they had seen the placard and/or posters. There was no specific feedback on the flyers, or font and layout of the information statement; however, as they did not appear to hinder the research processes, they were retained for the duration of recruitment.	Time required for research team to design materials and obtain HREC approval, plus cost for printing materials. Using posters, flyers and placards relied on frequent conversations with clinic staff, which was good for relationship building and required researchers to be sensitive and flexible regarding the needs and pressures of busy clinics.
Data collection and measurement				
Anticipated low literacy [1, 7]	Direct data collection	All data were designed for direct collection via face-to-face visits or phone interviews, unless visits/phone calls were not possible (see point below).	There were minimal missing data over time, e.g. proportions of missing data for the sensitive risk factors asked at enrolment (e.g. drug use, domestic violence) ranged from 0 to 5% per item, suggesting that items were acceptable and understandable. Further, we retained high proportions of the originally-enrolled sample to 2 years (82.5%), suggesting that the data collection strategy supported retention. Note: women with insufficient English to participate in face-to-face interviews were excluded from the trial (667/9511 (7%) screened in clinic waiting rooms) [12]	Research findings may not generalize to women with insufficient English to participate. In addition, women with low literacy may be overrepresented in the 923/6977 (13%) women who declined to complete the recruitment survey [12].
Blocked phone numbers would not have identified the caller	Phoning and texting from unblocked, active mobile/cell numbers	The clinic staff and intervention workforce advised the field researchers to use unblocked phone numbers to contact participants instead of the standard, institute-based blocked numbers. This allowed participants to identify the caller in advance. The mobile/cell phones also allowed for conversations with participants via text message, which the research team relied on heavily for booking assessments. Similarly, researchers used text messages to follow-up unanswered calls (see below) instead of leaving voicemails, because the cost of accessing voicemails may have deterred some participants from listening to them.	There are no data to describe whether these strategies supported retention; however, our researchers relied entirely on mobile/cell phone and text message contact.	Costs for purchasing mobile phones and plans, which were more expensive than using the institute-based landline phones.
Challenges in contacting participants via phone call				
Challenges in contacting participants	Enough contact attempts to give participants opportunity to provide data without causing bother	For each assessment, we implemented a 4–5 month window for data collection before a participant was ‘lost to follow-up’ for that time point. A protocol specifying a maximum number of contact attempts was trialed, ranging from as few as 8 contact attempts per assessment to more than 30. For this RCT, a contact attempt referred to a researcher’s attempt to contact a participant including interaction between the researcher and participant that followed the attempt directly, e.g. texts or phone calls back and forth between the researcher and participant in 1 day, or a text sent by a researcher on 1 day which the participant replied to the next day. Contact attempts included emails but excluded the standard reminder phone calls/texts, postal surveys, and Facebook messages that are described below.	During data collection, the field researchers found that 8 contact attempts were too few to maximize data collection with the hardest-to-reach participants, but were concerned that 20–30 attempts bothered participants. By the end of the 2 year follow-up, the team had established a contact process that allowed for approximately 16 contact attempts per assessment period. This revised protocol was supported by the contact notes; these were retrospectively analyzed for a random selection of 100 participants approached for the 2 year follow-up. For this random sample, an average of 3 contact attempts was needed to complete the assessment (range 1–20). 68 were completed in 1–3 attempts; 16 in 4–6 attempts; 4 in 7–10 attempts; and 4 in 11–20 attempts. 8/100 were not contactable. There was no evidence of differences between trial arms in the number of contact attempts made.	HREC approval was required for protocol modifications. Employing a specific number of contacts (e.g. 16) meant researchers needed to target their efforts to optimize each attempt, e.g. calling at different days, different times, after hours, weekends etc., and this approach was designed by the research coordinator and then monitored collectively by the field researchers. Providing after hours/weekend options to participants also requires employing a research workforce who can work flexibly.
Minimizing burden of data collection, and anticipated loss-to-follow-up and withdrawals over time	Data linkage	Participants were invited to consent for linkage to additional data sources due for future collection, to minimize the burden of future data collection, and maximize the longitudinal data available where participants are lost to follow-up. This included visits to the usual child and family health service (CFH, consent collected at baseline) and the Tasmanian Kindergarten Development Check (TKDC), the Victorian School Entrant Health Questionnaire (SEHQ) and the National Assessment Program – Literacy and Numeracy (NAPLAN) (consent collected at 1 year).	711/722 (98.5%) of enrolled participants consented to CFH service data linkage. 420/485 (86.6%) of enrolled Victorian participants consented to linkage with the SEHQ, and 197/237 (83.1%) of enrolled Tasmanian participants consented to linkage with the TKDC. 616/722 (85.3%) of all enrolled women consented to NAPLAN data linkage. These proportions are all higher than the proportion of participants providing data at 2 years (82.5%) and higher than the cohort who would be expected to complete assessments to school entry.	HREC approval was required for protocol modifications. There will be future staffing costs necessary to conduct the data linkage and analysis. There is a risk that the future managers of the state and national datasets will decline linkage requests; however, authors have already accessed linkage for SDQ and NAPLAN for other projects [13]. Furthermore, linkage is a fantastic opportunity for obtaining long-term, representative data on a diminishing cohort.
Retention and attrition				
Minimizing instances that participants fail to attend direct assessments	Reminders for direct assessments (data collection)	Participants’ plans changed day-to-day, so the research team employed several reminder processes. Text messages were sent on Mondays to all participants with a direct assessment scheduled in the week. A phone call was made the day before the visit to confirm and reschedule if necessary. Text messages were also sent the morning of assessments to confirm and reschedule if necessary.	There were no data to describe whether these strategies supported retention; however, anecdotally, they made it easier to book and rebook assessments as participants responded and initiated contact with researchers. For example, for the 596 women who provided data at 2 years, 119 (20%) rescheduled the follow-up assessment once or twice, and 13/596 (2%) rescheduled at least 3 times (78% completed the assessment without rescheduling), so field researchers needed to be flexible to families’ changing calendars and rebook assessments rapidly so as not to lose contact with the participant and momentum for the follow-up.	Costs for purchasing mobile phones and plans, which were more expensive than using the institute-based landline phones. Researcher time was required to develop templates for text messages (to convey meaning in a short message and avoid sending lengthy texts), plus relevant HREC approval.
	Reminders for phone assessments	Researchers texted before calling, or texted after a missed call, instead of leaving voicemail. This creates an opportunity for participants to text back with a suitable time to talk, or arrange a follow up visit by text.		
Anticipated loss-to-follow-up due to a participant’s changed contact details	Flexible data collection methods	Where the intended method of follow-up was not possible (e.g. face-to-face), participants were offered options including postal surveys or phone surveys to accommodate issues with their availability, new address interstate or overseas, low English proficiency, or personal preference.	Of 596 women who provided data at 2 years, 5 (0.8%) completed a postal survey and 19 (3.2%) completed a phone instead of the direct assessment.	Time was required to design assessment options and obtain HREC approval for protocol modifications, plus any printing and postage costs.
	Recording a variety of participant contact information	These were collected from participants and updated at every assessment where available: researchers recorded addresses, home phone numbers, mobile phone/cell numbers, secondary phone numbers (e.g. work), email addresses and best time to contact participants during the we ek or day.	During the period from enrolment to the 2 year follow-up (actual or due), of the 722 enrolled participants, 31% moved once, 18% moved 2–3 times, and 2% moved at least 4 times (49% did not move). There was no evidence of differences between trial arms in number of address changes. With regards to primary mobile phone/cell number changes, for the 722 enrolled participants, 23% changed their primary mobile/cell number once, 13% changed it 2–3 times, and 2% changed it at least 4 times (63% did not change it). Note, proportions add to > 100% because of rounding.	The cohort was highly mobile. These data underestimate the true frequency of changes because they only represent those made known to the research team, and the address and phone changes for the participants lost to follow-up are not represented. Similarly, we present the number of changes to the primary mobile/cell phone number because this was the main means for contact; however, there were many other contact changes for landline (home, work) and other (partner, parent) phone numbers. Given the frequency of changes, recording a range of contact information was crucial for retention.
	Recording alternate contacts who will know a participant’s updated details	Two alternate contacts collected and updated at every assessment where available: name, relationship, address, email, home and mobile/cell numbers.	Alternate contact use examined for a random selection of 100 participants approached for the 2 year follow-up (same sample as above). It was used once for 6 participants, and for all 6 (100%), the 2 year assessment was completed. All 6 of these participants were in the usual care group (none were in the intervention group). While the sample size is small, it suggests that alternate contacts were required more for the usual care group than the intervention group. This is likely because intervention families had more constant contact with the research team through the frequent, regular visits with intervention nurses, where usual care families did not.	This cohort was highly mobile and, anecdotally, the participants often told researchers that they went in and out of contact with family members and partners, so it was important collect details for more than one alternate contact. The alternate contacts also relayed similar changes in their relationship with the participants to the researchers.
	Permission to obtain new contact details from government social support program	Introduced at Baseline and completed at the 1 year assessment, participants were invited to provide consent for the research team to obtain their updated contact details (where they were not contactable through any other means) from a government social support program (“Centrelink”), which administers universal benefits such as the child care rebate plus means-tested benefits like pensions and prescription subsidies.	Of 697 women invited to consent to this linkage, 604 (87%) agreed. By the end of the 2 year follow-up (December 2016), we had contacted the government department 5 times to request updated participant contact details. The time taken to receive the updated data from the department ranged from 8 days to 7 months. Overall (totaled across the 5 contact points with the department), we requested updated contact details for 81 participants; of these, we received new details for 51 (63%), including 35 (69%) phone numbers, 32 (63%) addresses, and 20 (39%) email addresses. Of the 51 participants, we were subsequently able to contact 23 (45%).	Time was required to design the consent processes, and obtain HREC and government approvals. As this was a new type of collaboration for the government department, and the group responsible was restructured during the research, the process (to apply for and receive data) was time consuming and inefficient. However, subsequent requests for the RCT’s extended follow-up in 2017 (see Protocol) were far more efficient, with an average time of less than 2 weeks from request to receipt of updated contact data.
	Using social media to identify participants	During the 2 year assessment and after the governing HREC had implemented guidelines around using social media, researchers started searching for participants who we had lost contact with using Facebook in May 2016. Those who were identifiable from their name, age, email address and location (and sometimes family members including children) on public pages were contacted by direct messenger using a standardized message about the research project.	From May to December 2016, we searched for 107 participants on Facebook. We successfully identified 63 participants (59%) and messaged 61 (2 contacted the research team themselves). Of the 61 messaged, 35 (57%) saw the message (indicated by a Facebook function) and 11 (18%) responded by writing back. Responses included providing new contact details (46%), wanting to remain in the study (36%), or withdrawing (18%) from the study. Overall, of the 61 participants messaged, 20 (33%) were eventually contacted and 7 completed the 2 year assessment.	HREC approval was required for protocol modification. Time was required to create a project page, to search for and identify participants via public details, and send messages. There are substantial implications for confidentiality if participants of a research trial interact with Facebook page, so the project’s page was designed as a portal for messaging, and it was not possible for participants to interact with (e.g. like or comment on) the page.
Acknowledging participants’ contribution to the study	Annual newsletter	End of year newsletter sent to all enrolled participants to thank them for their participation, and to provide an update on the research, achievements, preliminary findings, staff biographies and student projects.	There were no data to describe whether newsletters supported retention; however, the research team considers it a way to thank participants and answer common participant questions. This type of feedback is also required by the governing HRECs.	HREC approval was required for protocol modifications. Time was required to design and collate newsletter, there were costs for printing.
	Remuneration for time needed for annual, face-to-face assessments	$30 vouchers for supermarket chains (for groceries only) were given to participants to thank them for each face-to-face (i.e. annual) assessment completed.	Participants in the aforementioned 2013 recruitment pilot study [8] were asked if remuneration would help them participate in the larger RCT; $20–$30 was the preferred voucher amount (preferred by 40% of participants; however, 21% of women reported that the amount did not matter. While we did not collect data on the usefulness of vouchers in this RCT, we know that participants used them because they commonly asked for them, and contacted the research team when vouchers needed activating or a researcher had run out of vouchers at a visit.	Substantial research cost. Appeared to be important for participant engagement.

*BRF* Brief risk facto survey, *HREC* Human Research in Ethics Committee, *SEIFA* Socio-Economic Indexes for Areas Index of Relative Disadvantage

^a^SEIFA calculated based on data for 1424 women who were eligible and 4135 who were not eligible due to missing data

The Table 1 lists the barriers and strategies. Those that were identified in the existing literature and implemented during the design phase are described with terms like “potential or anticipated”; otherwise, the barriers are those encountered during the trial. The Table 1 presents the findings according to 4 of the 5 research phases identified by Bonevski’s systematic review: [1] sampling; recruitment and gaining consent; data collection and measurement; and retention and attrition (intervention delivery is excluded as it was coordinated by a different workforce for the RCT). Where available, the number or proportion of women recruited or retained are used to evaluate the usefulness of strategies. Since assessment points (and therefore the research processes) were repeated multiple times, some processes are evaluated using data for one assessment point (most often the 2 year assessment as this was the primary outcome point and the furthest from recruitment). Data are provided for the complete sample where they were collected as discrete fields in the database and therefore easily exported and analyzed. Where information needed to be identified and extracted from the detailed descriptive participant notes, the data analysis was limited to a random sample of 100 participants selected using Stata (Intercooled Stata, v14·2 for Windows: College Station, TX, USA).

## Results

**Fig. 1:**
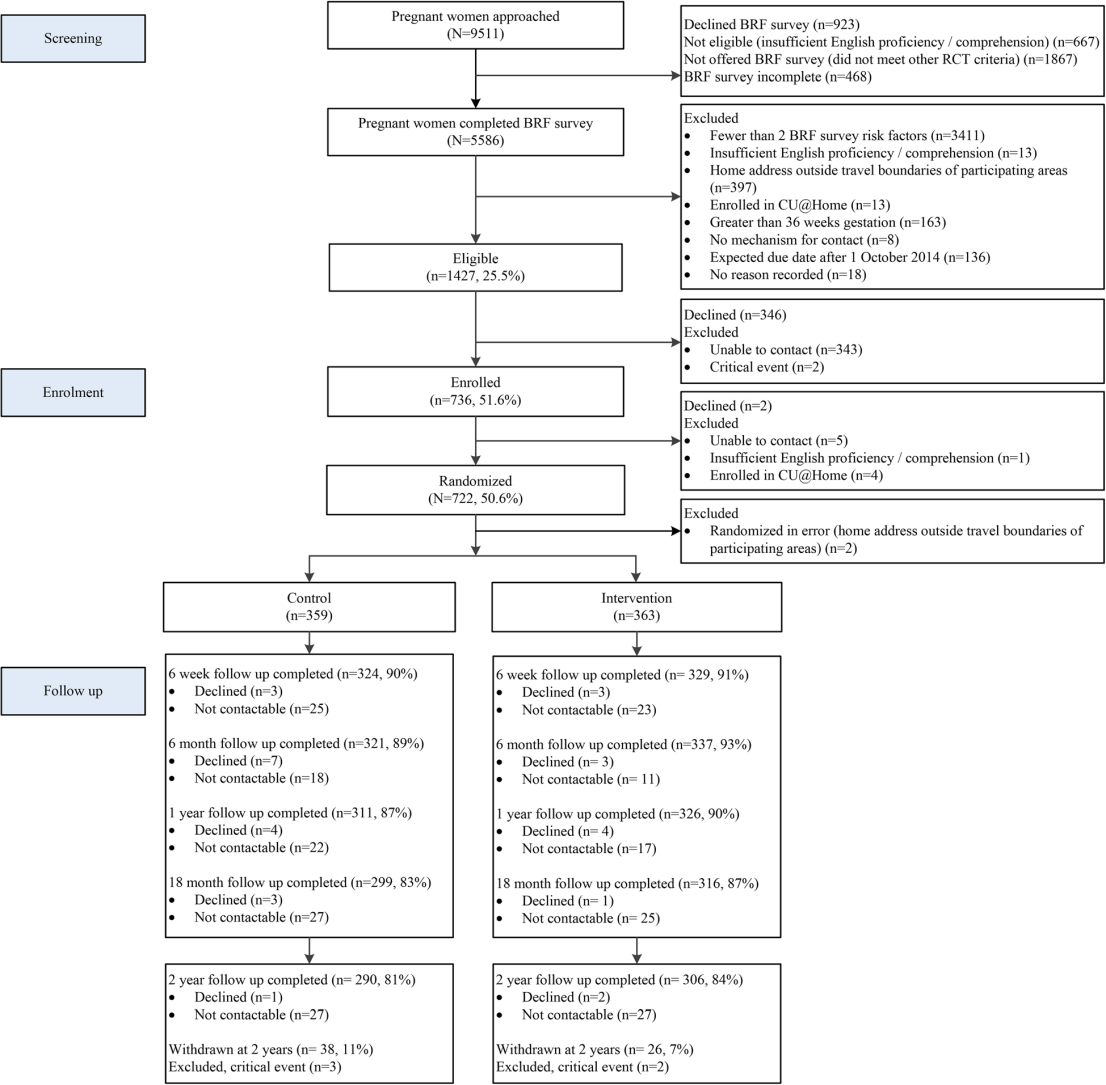
Participant flow from recruitment to the 2 year assessment

Of the 9511 pregnant women approached at clinics, 6977 were eligible and 5586/6977 (80.1%) completed the BRF survey (see Fig. 1). Of these, 1427 were eligible for the RCT, and the final enrolled cohort comprised 722 women experiencing high levels of adversity (see Additional file 2: Table S2), surpassing the necessary sample size. We maintained excellent retention and completion rates to child age 2 years (see Fig. 1): 90.4% completion and 1.5% cumulative withdrawal at 6 weeks; 91.1%/2.8% at 6 months; 88.2%/4.6% at 1 year; 85.1%/6.4% at 18 months, and 82.5%/8.9% at 2 years. The gaps between completion and cumulative withdrawal reflect the proportions of women who declined or were not contactable.

As described in the Table 1, most strategies appeared to support recruitment and retention. After recruitment started, new strategies required time from the research team to develop, obtain ethical approval (where necessary) and implement. Some changes also required additional funding, which ranged from minor (e.g. printing, postage costs) to substantial (e.g. salaries to extend the recruitment phase, funding participant remuneration). There was no evidence of differences between trial arms in the times families changed addresses or the number of contact attempts made toward completing an assessment. However, it was more common to reach out to the alternate contacts for usual care participants than for intervention participants (see Table 1). This is likely because intervention families had more frequent contact with the research team through their regular visits with intervention nurses, whereas usual care families did not. There was no evidence of differential attrition between trial arms by the 2 year follow-up assessment.

Several contextual factors supported recruitment and retention. We hired researchers who were highly skilled in building relationships with the participating women; provided rigorous researcher training in the project’s standard operating procedures; and employed a research coordinator (alongside the research manager) who was responsible for monitoring recruitment and retention by being the central point of contact for researchers, families, clinics and other contributors (e.g. Centrelink representatives). With this central role, it was possible to implement protocol changes relatively rapidly (dependent on other approvals such as HREC), obtain feedback and evaluate their usefulness.

## Discussion

Using a quality improvement approach to evaluate and optimize the research processes for a longitudinal, health services RCT resulted in the recruitment of a large cohort of pregnant Australian women experiencing adversity, and a high proportion of women retained to child age 2 years. Two main factors supported these successful research processes. First, the RCT had sufficient funding for the substantial research costs, which included salaries of skilled researchers employed for sufficient hours to conduct in-person research, plus researcher travel and participant remuneration. Second, we had flexibility as far as possible within a research paradigm to make continual updates and changes to the research protocol, which relied on collaborations with hospital clinic staff and ethics committees to approve and implement protocol amendments quickly.

A consequence of engaging and maintaining a large cohort is that it provides sufficient power to detect small between-group differences. This also allows for ongoing research follow-up beyond 2 years, since the sample remains large enough to meaningfully analyze long-term effects. We are confident that our efforts to obtain consent for data linkage will prove fruitful for long-term follow-up; the CFH data have already been used to conduct a cost evaluation of the intervention. Strategies in this study were implemented together (and are not mutually exclusive). This means we can only report recruitment and retention data corresponding to the relevant time period, rather than as a direct outcome of a specific strategy. For some strategies, we have no supporting data or only anecdotal reports. A formal evaluation of strategies (e.g. using a RCT design rather than a descriptive review) could provide evidence on effectiveness [1]. However, our intention was to evaluate whether a flexible quality improvement process, that implemented multiple strategies, could achieve high recruitment and retention.

Traditional data collection processes with large cohorts, or those experiencing more advantage, use postal or online surveys and minimal reminder processes. Given the number and types of contact needed to retain participants in right@home, traditional process would have failed to adequately engage and retain the cohort. Consistent with our findings, previous studies have demonstrated strong support for person-to-person (e.g. phone or direct) data collection and flexible data collection methods, with mixed evidence for the usefulness of promotional materials and readability [7]. Almost all other studies that have examined the effect of remuneration, including incentives, report that remuneration supports participant engagement [1]. These studies emphasize the need to keep remuneration clear of coercion, yet, remuneration remains controversial even though the costs incurred by participants are well-established. As Swanson noted over 20 years ago, [5] funding agencies need to recognize both the considerable cost of research with minority groups as well as the importance of participant remuneration. Finally, we found that strategies that minimized “barriers to entry”, such as recruiting in clinic waiting rooms, and booking assessments on-the-spot with women or by text messages, as well as all strategies to maintain up-to-date contact details for participants, were important features supporting retention and data collection.

These findings are relevant for community-based research that focuses on minority groups. The findings emphasize the need for increased funding and timeframes for recruitment and retention when compared with research with cohorts experiencing less adversity. Research with individuals experiencing adversity should be considered for the required level of funding by granting bodies when competitive funding is reviewed and awarded [5]. Future studies could evaluate strategies in a more systematic way by obtaining feedback from participants via single or simple questions asking for opinions on processes asked in-person at assessments or by text or email. In addition, it is important to increase the transparency around the true cost of the activities that make up this type of research; for example, how many researcher hours are needed, per participant, to complete enrolment or follow-up assessments.

## Conclusion

Research that includes women and families experiencing adversity is crucial for designing health services to meet the needs of those least likely to access a service but most likely to benefit. This is only possible with sufficient funding and flexible research processes. Taking a quality improvement approach and assessing each research process in detail can support high recruitment and retention. By increasing the transparency of research processes, researchers can argue the case for adequately resourcing research to ensure robust estimates of effectiveness. This will ultimately improve the quality and reach of health services for those who need them most.

## Supplementary information

**Additional file 1: Table S1.** Eligibility criteria for participation in the right@home RCT [4].

**Additional file 2: Table S2.** Brief Risk Factor (BRF) Survey item frequencies for RCT cohort (*N* = 722).

## Data Availability

The datasets generated and/or analyzed during the current study are not publicly available due to ongoing analyses.

## Abbreviations

BRF: Brief risk factor survey
CFH: Child and Family Health
HREC: Human Research in Ethics Committee
ISRCTN: International Standard Randomized Controlled Trials Number
RCT: Randomized controlled trial
SEIFA: Socio-Economic Indexes for Areas (SEIFA) Index of Relative Disadvantage
