# Supplementary material for: Processes for engaging and retaining women who are experiencing adversity in longitudinal health services research
Source: BMC Health Serv Res. 2019 Nov 14;19:833. doi: 10.1186/s12913-019-4698-5 (PMC6854799; doi:10.1186/s12913-019-4698-5)
Supplement: Supplementary file 1 — Additional file 1: Table S1. Eligibility criteria for participation in the right@home RCT [4]. [file 12913_2019_4698_MOESM1_ESM.docx]

Additional Table 1: Eligibility criteria for participation in the right@home RCT[4]

| **Inclusion criteria** | **Exclusion criteria** |
| --- | --- |
| 1. expected due dates before 1 October 2014, 2. less than 37 weeks gestation, 3. sufficient English proficiency to verbally answer interview questions, 4. two or more of 10 risk factors identified by the self-reported brief risk factor survey: (i) poorer global health, (ii) a long-term illness, health problem or disability that limits daily activities; (iii) smoking; (iv) young maternal age; (v) not living with another adult; (vi) no support in pregnancy; (vii) significant stress and coping difficulties; (viii) low education; (ix) no person in the household who currently earns an income; and (x) never having had a job before, 5. home addresses were within the travel boundaries specified by the participating area. | 1. enrolled in the existing Tasmanian CU@Home program (a state based nurse home visiting program for 15-19-year-olds), 2. did not comprehend the recruitment invitation (e.g. had an intellectual disability such that they were unable to consent to participation, or had insufficient English to complete face to face assessments), 3. had no mechanism for contact (landline or mobile telephone, or email address), or 4. experienced a critical event that excluded their participation (e.g. termination of pregnancy, still birth, participant or child death). |
