# Supplementary material for: Processes for engaging and retaining women who are experiencing adversity in longitudinal health services research
Source: BMC Health Serv Res. 2019 Nov 14;19:833. doi: 10.1186/s12913-019-4698-5 (PMC6854799; doi:10.1186/s12913-019-4698-5)
Supplement: Supplementary file 2 — Additional file 2: Table S2. Brief Risk Factor (BRF) Survey item frequencies for RCT cohort (N = 722). [file 12913_2019_4698_MOESM2_ESM.docx]

Additional Table 2: Brief Risk Factor (BRF) Survey item frequencies for RCT cohort (N=722)

| **Adverse BRF Survey Items** | **RCT participants**  **N (%)** |
| --- | --- |
| Poorer global health | 521 (72.2) |
| Long-term illness | 170 (23.6) |
| Smoking | 237 (32.8) |
| Young maternal age (<23 years) | 193 (26.7) |
| Not living with an adult | 121 (16.8) |
| No support in pregnancy | 62 (8.6) |
| Anxious mood | 218 (30.2) |
| Education <Year 12 | 429 (59.4) |
| No household income | 236 (32.7) |
| Never had a job | 108 (15.0) |
| Total risk count (mean(SD), [range]) | 3.2 (1.3), [1-8] |
